# Research on an LEO Constellation Multi-Aircraft Collaborative Navigation Algorithm Based on a Dual-Way Asynchronous Precision Communication-Time Service Measurement System (DWAPC-TSM)

**DOI:** 10.3390/s22093213

**Published:** 2022-04-22

**Authors:** Lvyang Ye, Yikang Yang, Jiangang Ma, Lingyu Deng, Hengnian Li

**Affiliations:** 1 School of Electronic and Information Engineering, Xi’an Jiaotong University, Xi’an 710049, China; yely2019@stu.xjtu.edu.cn (L.Y.); jiangangma@stu.xjtu.edu.cn (J.M.); dengly0625@stu.xjtu.edu.cn (L.D.); 2 State Key Laboratory of Astronautic Dynamics, General Armament Department, Xi’an Satellite Control Center, Xi’an 710043, China; henry_xscc@mail.xjtu.edu.cn

**Keywords:** cooperative positioning, multi-aircraft, time synchronization, ranging, velocity measurement, LEO

## Abstract

In order to solve the collaborative navigation problems in challenging environments such as insufficient visible satellites, obstacle reflections and multipath errors, and in order to improve the accuracy, usability, and stability of collaborative navigation and positioning, we propose a dual-way asynchronous precision communication–timing–measurement system (DWAPC-TSM) LEO constellation multi-aircraft cooperative navigation and positioning algorithm which gives the principle, algorithm structure, and error analysis of the DWAPC-TSM system. In addition, we also analyze the effect of vehicle separation range on satellite observability. The DWAPC-TSM system can achieve high-precision ranging and time synchronization accuracy. With the help of this system, by adding relative ranging and speed measurement observations in an unscented Kalman filter (UKF), the multi-aircraft coordinated navigation and positioning of aircraft is finally realized. The simulation results show that, even without the aid of an altimeter, the multi-aircraft cooperative navigation and positioning algorithm based on the DWAPC-TSM system can achieve good navigation and positioning results, and with the aid of the altimeter, the cooperative navigation and positioning accuracy can be effectively improved. For the formation flight configurations of horizontal collinear and vertical collinear, the algorithm is universal, and in the case of vertical collinear, the navigation performance of the formation members tends to be consistent. Under different relative measurement accuracy, the algorithm can maintain good robustness; compared with some existing classical algorithms, it can significantly improve the navigation and positioning accuracy. A reference scheme for exploring the feasibility of a new cooperative navigation and positioning mode for LEO communication satellites is presented.

## 1. Introduction

At present, unmanned equipment has been widely used and the focus of attention in military and civilian applications. In addition to civilian unmanned transportation, disaster relief, and risk elimination, it also plays an important role in modern warfare. In particular, unmanned equipment was used in the war between Azerbaijan and Armenia, and has become an indispensable part of the military equipment of major countries around the world. However, with the rapid development of modern military equipment, the execution efficiency of individual manned/unmanned equipment (unmanned combat vehicles, unmanned aerial vehicles, unmanned boats, etc.) is often limited, and complex combat tasks cannot be completed properly. Cooperative positioning (CP) is a method for enhancing navigation and positioning among multiple participants. Through specific means of communication and fusing the relevant information of all participants, such as the position, velocity, and attitude, the cooperative navigation and positioning function is realized [1,2,3]. In aircraft formation flight, ship formation navigation, aircraft air rendezvous and docking, as well as in other applications, increased redundant backup, cost reductions, and improved accuracy and availability of positioning performance can be realized. CP is an important technical means for all components of a system to obtain high-precision spatiotemporal unified information [4,5,6,7]. In addition, with the rapid development of artificial intelligence (AI), autonomous driving, and 5G/6G technologies, people’s requirements for the accuracy of location services are increasing, especially in autonomous driving and drone formation flight performances; among them, collaborative navigation and positioning technology is an important supporting tool for safe driving and flying [8,9,10,11].

To meet the development needs of 6G, AI, and smart cities in the future, there is an urgent need for an absolute positioning and cooperative positioning system, as well as corresponding algorithms that can adapt to the future with high precision and low latency. In recent years, the low-earth orbit (LEO) constellation, with its unique advantages of low propagation delay, less link loss, and relatively strong signal power [12,13], is expected to be used in unmanned cooperative operations and autonomous driving in the future. In addition, airborne data link systems, such as the joint tactical information distribution system (JTIDS), have the integrated functions of communication, navigation, and identification, can be used by sea, land, and air motion carriers, and have interoperability in sea, air, and land coordinated operations. The JTIDS is a large-capacity, anti-jamming digital information distribution system [14]. The global navigation satellite system (GNSS) information and inertial navigation system (INS) information of two or more nodes are shared on one node by using the communication function of the airborne data link, and the measurement information between nodes is obtained by using the precise ranging and velocity measurement functions of ranging and velocity sensors. Then, the relative GNSS information, relative INS information, and measurement information are combined to realize the joint correction of multiplatform INS errors, and the optimal cooperative navigation information is obtained. On the one hand, the scheme has the advantages of a large amount of shared information and high frequency, especially the proprietary link of the system, which can ensure the large amount of information and high update rate shared between nodes [15]; on the other hand, due to the introduction of high-precision measurement information, the problem where cooperative navigation accuracy is overly dependent on GPS is well resolved.

Based on the LEO constellation, by analyzing the theoretical derivation of the clock bias cancellation system, reference [16] presents a single-satellite close-combined navigation and positioning scheme through clock bias elimination similar to the full-duplex (FD) system and altimeter-assisted navigation and positioning in challenging environments, which can effectively improve the performance of traditional GNSS multi-aircraft navigation and positioning, but the article does not give a specific clock bias elimination scheme. Reference [17] provides an alternative solution to the problem of GNSS signal availability in urban canyons based on V2X technology through dedicated short-range communication (DSRC), enabling vehicles in GNSS-denied environments to rely on other vehicles with sufficient GNSS signal vehicles to determine their position to achieve cooperative navigation and positioning, but this scheme is limited by the system it adopts, and is only suitable for short-distance cooperative navigation. Reference [18] developed a non-line-of-sight (NLOS) GNSS signal detection algorithm for the problem of multipath delay and signal interruption in collaborative navigation systems in urban environments, and proposed an anti-multipath network algorithm and anti-multipath cooperative extended Kalman filter (EKF) technology; however, the solution based on traditional GNSSs obviously cannot meet the technical requirements of future integrated communication and navigation (ICN) of low cost and low transmission delay in the future. Reference [19] studied a tightly coupled GNSS/ultrawideband (UWB)/INS cooperative positioning scheme utilizing the robust Kalman filter (RKF) supported by V2I communication that uses the distance measurements of UWB cells transmitted between terminals as the augmented input for the observations. The GNSS observation equation composed of pseudorange and Doppler measurements is transformed using the UWB distance input, and the updated observation equation is processed into a tightly coupled GNSS/UWB/INS combination using an adaptive RKF positioning equation. This scheme provides better positioning accuracy and improves the usability of the system in GNSS-denied environments, but this scheme relies too much on traditional GNSS. As such, in challenging environments, this scheme may cause large positioning errors or even cause the algorithm to fail.

In view of the shortcomings and deficiencies of the above cooperative navigation system and its algorithms, we propose a multi-aircraft cooperative navigation and positioning algorithm for the LEO constellation in the dual-way asynchronous precision communication–time–service measurement (DWAPC-TSM) system. This algorithm can effectively complete high-precision ranging and time synchronization, which helps to ensure clock bias elimination and precise ranging for coordinated navigation and positioning. By adding relative ranging and velocity measurement values and then through multi-aircraft tightly integrated collaborative navigation filtering, the capability of inter-aircraft collaborative navigation and positioning can be achieved at the same time. The subsequent arrangement of the article is as follows: Section 2 describes the principle and algorithm structure of the DWAPC-TSM system, and analyzes the ranging accuracy and time synchronization error, which is the theoretical support for DWAPC-TSM cooperative navigation and positioning. In Section 3, the principle and processing flow of the DWAPC-TSM system cooperative navigation and positioning algorithm and the corresponding state equation and observation equation are established, and the influence of the distance range of the aircraft on the observability of the satellite is analyzed. In Section 4, simulation analysis is given for an altimeter and a system without an altimeter assistance, two special formation flight configurations, navigation and positioning performance under different relative measurement accuracy, and the universality of LEO constellation, that support our theory. In Section 5, we compare our algorithm with other typical algorithms to discuss the advantages and disadvantages of our proposed algorithm, and highlight the improvement direction for the follow-up work. The last section of the article gives our conclusions and directions for future research.

## 2. Time Synchronization and Ranging Scheme Based on a Dual-Way Asynchronous Precision Communication-Time Service-Measurement System (DWAPC-TSM)

The dual-way asynchronous precision communication–time–service measurement system (DWAPC-TSM), which includes a dual-way asynchronous precision communication-timing system and a dual-way asynchronous precision ranging system, is based on the principle of dual one-way ranging (DOWR) [20]. The dual-way ranging and time synchronization system of DOWR is installed in the signal transmitter and receiver of the dual ranging terminals in the system. In this ranging method, the transmitting path of the ranging signal is basically the same as the receiving path, except that the directions are opposite; thus, the ranging mode can minimize the influence of the propagation path error in the ranging process, and the clock bias can be eliminated by bidirectional measurement in order to achieve precise ranging and time synchronization between the two terminals. Although the equipment delay error is the main factor that affects the measurement accuracy of the system, the measurement accuracy of the DOWR method can be improved by measuring and calibrating the delay of the transceiver equipment in the dual-way ranging and time synchronization system [21]. In addition, the distance between most terminals near the ground is generally not large, usually within a few thousand kilometers [22]; therefore, the communication link between terminals follows the Consultative Committee for Space Data Systems (CCSDS) protocol. The CCSDS protocol is applicable, and the transmission frame structure design of version 3 can be referred to for the design of the transmission frame [23].

### 2.1. Dual-Way Asynchronous Precision Communication-Time Service-Measurement System (DWAPC-TSM) Principle and Algorithm

Based on the DOWR principle, we next present the dual-way asynchronous precision baseline measurement and asynchronous precision time synchronization (TWAP-BMandTS) principle and corresponding algorithm (TWAP-BMandTS-A) between DWAPC-TSM terminals, which will be introduced separately below.

#### 2.1.1. Principle of TWAP-BMandTS between Terminals

**Figure 1:**
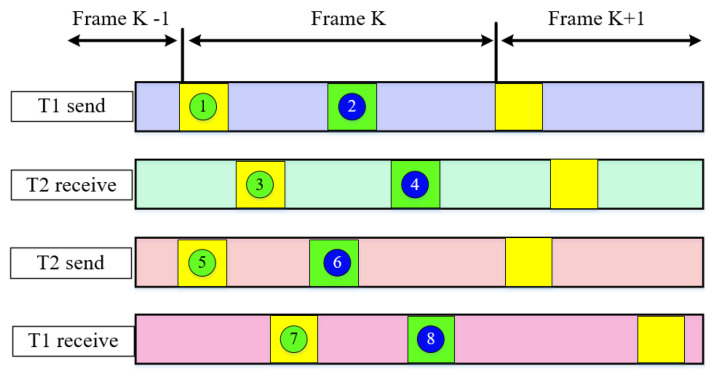
Transmission frame format and timing relationship of the DWAPC-TSM system.

**Figure 2:**
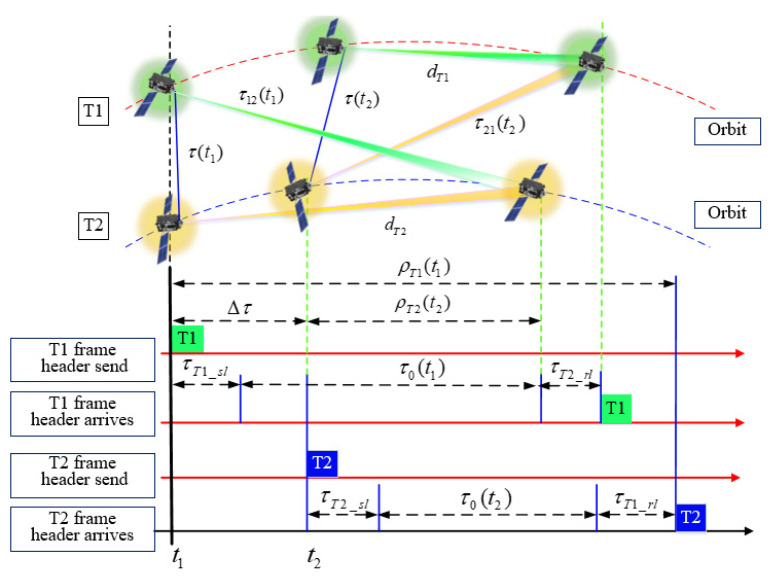
DWAPC-TSM system principle and timing relationship.

Without a loss of generality, we assume that each measurement terminal is equipped with a DWAPC-TSM unit. Here, we take two terminals as specific research targets, and name them T1 and T2. The two terminals send DWAPC-TSM-related frame information to each other independently and mutually. The baseband frequency and carrier transmission frequency used locally are generated by a local frequency synthesizer (LFS), and there is no attached constraint relationship between the two sides. The two terminals T1 and T2 independently use their respective local reference clock, local pseudorange information, received local reference clock, and local pseudorange information transmitted by the other party to adjust the calculated distance, time synchronization error, sampling interval (time), and other measurement information between terminals T1 and T2. The specific description of the DWAPC-TSM system is shown in Figure 1 and Figure 2.

The relevant parameter in Figure 1 are defined as follows:

① is the DWAPC-TSM system transmission frame synchronization code sent by terminal T1;

② is the local pseudorange measurement value of terminal T1;

③ is the DWAPC-TSM system transmission frame synchronization code received by terminal T2;

④ is the local pseudorange measurement value of terminal T1 in the DWAPC-TSM system transmission frame sent by terminal T1 and received by terminal T2;

⑤ is the DWAPC-TSM system transmission frame synchronization code sent by terminal T2;

⑥ is the local pseudorange measurement value of terminal T2;

⑦ is the DWAPC-TSM system transmission frame synchronization code received by terminal T1;

⑧ is the local pseudorange measurement value of terminal T2 in the DWAPC-TSM system transmission frame sent by terminal T2 and received by terminal T1, and K is the length of the spreading code (number of chips).

The parameters in Figure 2 are defined as follows:

(1) $\rho_{T1}(t_{1})$: the local pseudorange obtained by sampling the T1 DWAPC-TSM system frame header of the terminal at $t_{1}$ (which can be converted to an equivalent time value);
(2) $\tau_{T1}{{}_{\_}}_{sl}$: the terminal T1 sending delay;
(3) $\tau_{T1\_rl}$: the terminal T1 receiving delay;
(4) $\tau_{0}(t_{1})$: the transmission delay of radio waves between the antenna phase centers of terminal T1 and terminal T2 at the time of $t_{1}$;
(5) Δτ: the calculated value of the time difference, that is, the clock bias (bias in timing) between terminals T1 and T2 at time $t_{1}$.
(6) $\tau (t_{1})$: taking the clock of terminal T1 as a reference, the distance between terminal T1 and terminal T2 is delayed at the start of the transmission time slot of $t_{1}$;
(7) $\tau_{12}(t_{1})$: the spatial propagation delay for the signal transmitted by terminal T1 at time $t_{1}$ to reach terminal T2;
(8) $d_{T1}$: the motion distance vector of terminal T1 within the propagation delay;
(9) The meanings of $\rho_{T2}(t_{2})$ (which can be converted to an equivalent time value), $\tau_{T2\_rl}$, $\tau_{T2\_sl}$, $\tau_{0}(t_{2})$, $\tau (t_{2})$, $\tau_{21}(t_{2})$ and $d_{T2}$ are similar to the above parameter definitions, and will not be described here.

#### 2.1.2. TWAP-BMandTS Algorithm (TWAP-BMandTS-A) Construction between Terminals

Now let $\tau_{T1T2}=\tau_{T1\_sl}+\tau_{T2\_rl}$, $\tau_{T2T1}=\tau_{T2\_sl}+\tau_{T1\_rl}$; according to the DOWR distance and clock bias calculation of equation [20], we can obtain

(1) $$\{\begin{array}{l} \rho_{T1}(t_{1})=\tau_{T2\_sl}+\tau_{0}(t_{2})+\tau_{T1\_rl}+\Delta \tau \\ \rho_{T2}(t_{2})=\tau_{T1\_sl}+\tau_{0}(t_{1})+\tau_{T2\_rl}-\Delta \tau \end{array}$$

According to Figure 2, the DOWR timing relationship between terminals T1 and T2 can be obtained as follows:

(2) $$\{\begin{array}{l} b=\frac{1}{2}[(\rho_{T1}(t_{1})+\rho_{T2}(t_{2}))-(\tau_{T1T2}+\tau_{T2T1})]c\\ \Delta \tau =\frac{1}{2}[(\rho_{T1}(t_{1})-\rho_{T2}(t_{2}))+(\tau_{T1T2}-\tau_{T2T1})] \end{array}$$

where, *b* is the calculated distance; *c* is the speed of the electromagnetic wave; and $b=\tau_{0}c$.

In the case where the carrier tracking loop and code tracking loop of the terminal de-spreading/demodulation unit are well locked on the received signal, terminal T1 can calculate the pseudorange (time delay) measurement between the synchronization time of the local transmission frame and the frame synchronization time of receiving the transmission frame of terminal T2 (similar for terminal T2), namely [24]:

(3) $$\rho_{T1}(t_{1})=K_{T1,T2}PT_{b}-T_{b}\times \left(K_{T2,T1}P+S_{T2,T1}+\frac{M_{T2,T1}+\frac{N_{T2,T1}}{2^{r}}}{L_{\mathrm{PRN}}}\right)c$$

In Equations (1)–(3), $K_{T1,T2}$, $S_{T2,T1}$, $M_{T2,T1}$, and $N_{T2,T1}$ are the epoch information stored at the corresponding sampling time; $T_{b}$ is the corresponding bit period; $L_{\mathrm{PRN}}$ is the number of bits of the spread spectrum code; *P* is the frame number of bits; and *r* is the bit width of the digitally controlled oscillator (DCO) register. In Equation (3), the first item is the clock face reference time of the reference frequency used locally by the terminal. The second item represents the sending time when the signal sent from terminal T2 is sampled by the leading edge of the synchronization code clock in the local terminal T1 (DWAPC-TSM system sending frame) based on the local clock face time of terminal T1. Figure 1 and Figure 2 and Equations (1)–(3) show that, in the DWAPC-TSM system, both terminals need to send and receive pseudorange information of their own and to each other for calculation.

According to radio ranging theory and microwave communication theory [25,26,27], after analysis and derivation, the equations for definitions the ranging and clock bias of the DWAPC-TSM system can be obtained as follows:

(4) $$\left\{ \begin{array}{l} \Delta t=0.5\cdot [\rho_{T1}(t_{1})-\rho_{T2}(t_{2})-2\cdot \Delta \tau_{T1T2\_sl}(t_{2})+\tau_{\mathrm{drift}-}]+(\Delta \tau -\nabla \tau_{\Delta f_{T1T2+}}+\nabla \tau_{\tau -})\\ \Delta \tau_{T1T2}(t_{1})=0.5\cdot [\rho_{T1}(t_{1})-\rho_{T2}(t_{2})+\tau_{\mathrm{drift}-}]+(\Delta \tau -\nabla \tau_{\Delta f_{T1T2-}}+\nabla \tau_{\tau -})\\ \tau_{0}(t_{1})=0.5\cdot [\rho_{T1}(t_{1})+\rho_{T2}(t_{2})-\tau_{\mathrm{drift}+}]-(\Delta \tau -\nabla \tau_{\Delta f_{T1T2-}}+\nabla \tau_{\tau +}) \end{array}\right.$$

Equation (4) can be used for the time synchronization of DWAPC-TSM system channels. In the equation, $\Delta t=t_{2}-t_{1}$ is the sampling time interval of the two terminals; $\Delta \tau_{T1T2\_sl}(t_{2})=\tau_{T1\_sl}(t_{1})-\tau_{T2\_sl}(t_{2})$ is the arithmetic difference between the local transmission frame epoch time sampled by terminal T1 and the local transmission frame epoch time sampled by terminal T2 at time $t_{2}$; $\tau_{T1\_sl}(t_{1})$ and $\tau_{T1\_sl}(t_{2})$ are the terminal local epoch reference times sampled by terminal T1 and terminal T2 at times $t_{1}$ and $t_{2}$, respectively; and $\tau_{\mathrm{drift}+}$ and $\tau_{\mathrm{drift}-}$ are the corresponding combined drifts, and the error can usually be less than 0.1 ns after calibration [22]. In addition, the following relationships can be obtained [27]:

(5) $$\left\{ \begin{array}{l} \Delta \tau =0.5[\tau_{0}(t_{1})-\tau_{0}(t_{2})]=\int_{t_{1}}^{t_{2}}\frac{v_{ra}(t)}{c}dt\\ \nabla \tau_{\tau +}=\nabla \tau_{\tau -}=0.5\\ \nabla \tau_{\Delta f_{T1T2+}}=\frac{1}{2}\int_{t_{{}_{1}}}^{t_{{}_{2}}}\frac{f_{re\_T1}-f_{0}}{f_{0}}dt=\int_{t_{{}_{1}}}^{t_{{}_{2}}}\frac{\Delta f_{re\_T1}}{f_{0}}dt\\ \nabla \tau_{\Delta f_{T1T2-}}=\frac{1}{2}\int_{t_{{}_{1}}}^{t_{{}_{2}}}\frac{f_{re\_T2}-f_{0}}{f_{0}}dt=\int_{t_{{}_{1}}}^{t_{{}_{2}}}\frac{\Delta f_{re\_T2}}{f_{0}}dt \end{array}\right.$$

where $v_{ra}(t)$ is the relative velocity between the two terminals; $\tau_{0}(t_{1})$ and $\tau_{0}(t_{2})$ are the spatial distances between the antenna phase centers of the two terminals at times $t_{1}$ and $t_{2}$, respectively (represented by the radio wave transmission delay, unit: ns); $f_{re\_T1}$ and $f_{re\_T2}$ are the true values of the clock frequency of terminal T1 and terminal T2 of the DWAPC-TSM system unit; and $f_{0}$ is the nominal value of the code clock frequency of the terminal’s local DWAPC-TSM system unit.

According to Equation (5) combined with reference [27], we can obtain:

(6) $$\left\{ \begin{array}{l} |\Delta \tau |\leq \alpha |\Delta t|\begin{array}{cccc} & & & \left(\alpha =\underset{t}{\max|}\frac{1}{2}\frac{v_{ra}(t)}{c}|\right) \end{array}\\ |\nabla \tau_{\tau +}|\leq \beta \tau_{0}\begin{array}{cccc} & & & \begin{array}{cc} & \left(\tau_{0}=|\tau_{0}(t)|\right) \end{array} \end{array}\\ |\nabla \tau_{\tau -}|\leq \beta \tau_{0}\begin{array}{cccc} & & & \begin{array}{cc} & \left(\tau_{0}=|\tau_{0}(t)|\right) \end{array} \end{array}\\ \left|\nabla \tau_{\Delta f_{T1T2+}}|\leq \gamma |\Delta t\right|\\ \left|\nabla \tau_{\Delta f_{T1T2-}}|\leq \gamma |\Delta t\right| \end{array}\right.$$

According to Equations (4) and (6), the discussion is as follows:

(1) The relative velocity of the two terminals is small. Taking satellites and aerial vehicles as examples, the moving velocity of artificial satellites is approximately 7.9 km/s, and the moving velocity of general aircraft is usually between approximately Mach 1 and several times the speed of sound. Here, we consider $v_{ra}(t)|\leq 10$ km/s, that is, $\alpha \leq 1.67\times 10^{-5}$.
(2) Using an ultra-stable crystal oscillator or atomic frequency, the accuracy/stability parameter $\beta \leq 1\times 10^{-11}$ [28];
(3) Δt is not more than one transmission frame period, and can be accurately measured and converged to 0 after time synchronization adjustment;
(4) The scale of the terminal topological configuration is not too large, and the terminals are generally distributed in the range of several thousand kilometers. Here, we consider that $c\tau_{0}(t)\leq 3000$ km, that is, τ≤0.0167, and the algorithmic model error of the terminal baseline measurement, clock bias measurement, and sampling interval measurement is related to the product of β and $\tau_{0}$, $\beta \tau_{0}\leq 0.167$ ps @ $\beta \leq 1\times 10^{-11}\;\&\;c\tau_{0}(t)\leq 3000$ km.

Equations (4) and (6) show that when the sampling time interval is Δt=0, the model error caused by other factors is ≤0.1 ps (equivalent to a baseline measurement error of approximately 0.03 mm).

### 2.2. Time Synchronization and Ranging Error Analysis of TWAP-APBMandAPT-A

Taking the relative distance measurement between terminal T2 and terminal T1 as an example, and setting the difference between the true frequency values of terminal T2 and terminal T1 to be $\Delta f_{re}=f_{re\_T2}-f_{re\_T1}$, the pseudorange measurement deviation caused by the change in the clock bias is [29]:

(7) $$\Delta \rho =\frac{\Delta f_{re}}{f_{0}}\times \Delta \tau \times c$$

Taking the clock reference frequency $f_{0}=10.23$ MHz, by measuring the clock bias of terminal T1 and terminal T2 to ensure time synchronization, it is easy to control the transmission time difference of the measurement information of terminal T1 and terminal T2 within 0.1 ms, that is, Δτ = 0.1 ms. To ensure Δρ < 0.01 mm, the requirements for the relative frequency difference $\Delta f_{re}/f_{0}$ between terminal T2 and terminal T1 are:

(8) $$\frac{\Delta f_{re}}{f_{0}}=\frac{\Delta \rho }{\Delta \tau \times c}\leq 3.33\times 10^{-10}$$

Using atomic clocks with accuracy and stability better than $1.0\times 10^{-10}$ (such as: rubidium clocks, with an accuracy and a stability of $\;10^{-12}$, cesium clocks, with an accuracy and a stability of $10^{-13}$, and hydrogen clocks, with an accuracy and a stability of $\;10^{-15}$) as the reference, the frequency of terminal T1 and terminal T2 can meet the above requirements.

Within the Δτ clock bias, the relative movement between the terminals, that is, the change in the relative distance (baseline), also produces the deviation of the distance measurement and the error of the time comparison. Taking the baseline measurement of two terminals as an example, the ranging error caused by the baseline change within Δτ is:

(9) $$\Delta b=b_{T1}-b_{T2}=\int_{\Delta t}v_{ra}(t)dt={\bar{v}}_{ra}\cdot \Delta \tau$$

where ${\bar{v}}_{ra}$ is the average value of $v_{ra}(t)$ within Δτ. With Equation (2), the baseline and clock biases calculated by terminal T2 and terminal T1 are:

(10) $$\{\begin{array}{c} b_{T1}=\frac{1}{2}[(\rho_{T1}(t_{1})+\rho_{T2}(t_{2}))-(\tau_{T1T2}+\tau_{T2T1})]c+\frac{1}{2}{\bar{v}}_{ra}\cdot \Delta \tau \\ b_{T2}=\frac{1}{2}[(\rho_{T1}(t_{1})+\rho_{T2}(t_{2}))-(\tau_{T1T2}+\tau_{T2T1})]c-\frac{1}{2}{\bar{v}}_{ra}\cdot \Delta \tau \end{array}$$

(11) $$\left\{ \begin{array}{l} \Delta \tau_{T1}=\frac{1}{2}[(\rho_{T1}(t_{1})-\rho_{T2}(t_{2}))+(\tau_{T1T2}-\tau_{T2T1})]-\frac{1}{2}\frac{{\bar{v}}_{ra}\cdot \Delta \tau }{c}\\ \Delta \tau_{T2}=\frac{1}{2}[(\rho_{T1}(t_{1})-\rho_{T2}(t_{2}))+(\tau_{T1T2}-\tau_{T2T1})]+\frac{1}{2}\frac{{\bar{v}}_{ra}\cdot \Delta \tau }{c} \end{array}\right.$$

The above equation shows that, due to the clock bias Δτ, the baseline calculation error and the time difference (that is, the clock bias) calculation error caused by the relative motion between terminal T2 and terminal T1 are proportional to Δτ and ${\bar{v}}_{ra}$: when the two terminals are relatively stationary, that is, when $v_{ra}(t)$ = 0, the clock bias will not cause measurement error; when the clock bias between terminals is 0, that is, when Δτ = 0, the relative motion between terminals will not cause measurement errors. Assuming a measurement refresh rate of 0.1 s, the maximum value of the resolvable clock bias, $\Delta \tau_{s}$, for the ranging-time comparison is 0.1 s. Taking satellite and aircraft as an example, the relative velocity between terminals is $\left|v_{ra}(t)\right|$ < 10,000 m/s, therefore, for the mean value of $v_{ra}(t)$ in $\Delta \tau_{s}$, $\left|v_{ra}(t)\right|$ < 10,000 m/s. Taking terminal T2 as an example (terminal T1 is similar), when the clock bias is not adjusted before the time comparison, the baseline calculation error and clock bias calculation error are:

(12) $$\left\{ \begin{array}{l} \delta b_{1}=\left|0.5{\bar{v}}_{ra}\cdot \Delta \tau_{s}\right|\leq 500\;m\\ \delta \Delta \tau_{1}=\left|-\frac{0.5{\bar{v}}_{ra}}{c}\cdot \Delta \tau_{s}\right|\leq 1.67\;\mu s \end{array}\right.\begin{array}{cc} & \left(\left|{\bar{v}}_{ra}\right|=10,000\;m/s,\left|\Delta \tau_{s}\right|=0.1\;s\right) \end{array}$$

The above equation gives the maximum error between the baseline calculation and the time bias calculation when the clock bias is not adjusted and there is relative motion between terminal T2 and terminal T1. A time comparison is performed to calculate the clock bias and adjust the clock reference between terminal T2 and terminal T1. After this time synchronization, the residual clock bias between the terminals is equal to $\delta \Delta \tau_{1}$. Let $\Delta \tau_{s}$ = $\delta \Delta \tau_{1}$ (at this time, $|\Delta \tau_{s}|=\delta \Delta \tau_{1}<$ 10 μs). By performing the second calculation, we can obtained:

(13) $$\left\{ \begin{array}{l} \delta b_{2}=\left|0.5{\bar{v}}_{ra}\cdot \Delta \tau_{s}\right|\leq 0.05\;m\\ \delta \Delta \tau_{2}=\left|-\frac{0.5{\bar{v}}_{ra}}{c}\cdot \Delta \tau_{s}\right|\leq 0.167\;\mathrm{ns} \end{array}\right.\begin{array}{cc} & \left(\left|{\bar{v}}_{ra}\right|=10,000\;m/s,\left|\Delta \tau_{s}\right|=10\;\mu s\right) \end{array}$$

Let $\Delta \tau_{s}$ = $\delta \Delta \tau_{2}$ (at this time $|\Delta \tau_{s}|=\delta \Delta \tau_{2}<$ 1 ns); the third calculation is performed to obtain:

(14) $$\left\{ \begin{array}{l} \delta b_{3}=\left|0.5{\bar{v}}_{ra}\cdot \Delta \tau_{s}\right|\leq 5\;\mu m\\ \delta \Delta \tau_{3}=\left|-\frac{0.5{\bar{v}}_{ra}}{c}\cdot \Delta \tau_{s}\right|\leq 0.0167\;\mathrm{ps} \end{array}\right.\begin{array}{cc} & \left(\left|{\bar{v}}_{ra}\right|=10,000\;m/s,\left|\Delta \tau_{s}\right|=1\;\mathrm{ns}\right) \end{array}$$

The above equation shows that after the clock bias between the two terminals is adjusted to within 1 ns, the baseline measurement error and time synchronization error (that is, the calculation error of the clock bias) between the terminals due to the relative motion of terminal T2 and terminal T1 are small enough to be negligible, satisfying the requirements of ranging accuracy and clock bias measurement accuracy.

According to the relationship between the relative accuracy of the clock reference and the time bias, the time synchronization control error between terminal T2 and terminal T1 can be expressed as:

(15) $$\Delta \tau =\frac{\Delta f_{re}}{f}\times T_{C}$$

where $T_{C}$ represents the time interval of two clock synchronization controls. The time synchronization method obtains the clock bias between terminal T1 and terminal T2 through bidirectional measurement. For the requirements of the DWAPC-TSM system, the performance of the selected atomic clock, $\Delta f_{re}/f$, is better than $1.0\times 10^{-10}$, |Δτ| = 100 µs, is selected as the adjustment threshold, and the interval between two time synchronizations can be calculated: $T_{C}$ = 200,000 s. If the application requires a smaller clock bias between terminal T1 and terminal T2, the time synchronization interval can be reduced twice; for example, $T_{C}$ = 200 s, and a time adjustment accuracy of |Δτ| < 100 ns can be achieved. Through the DWAPC-TSM system, high-precision synchronization between terminals can be achieved; that is, the clock bias between terminals can be eliminated. Later, we will use this assumption; that is, we will consider that the clock bias between terminals achieves high-precision. Therefore, the clock bias will not be considered in the pseudorange observation equation, and the high-precision ranging function can be realized by this system.

## 3. Analysis of the Multi-Aircraft Cooperative Navigation Algorithm Based on the DWAPC-TSM System

The main idea of the cooperative navigation and positioning algorithm of the DWAPC-TSM system is to rely on the DWAPC-TSM system described in Section 2. The background of the problem is to rely on the currently updated and upgraded broadband LEO constellation for navigation and positioning in the traditional GNSS rejection environment, and we mainly focus on extremely challenging environments, especially canyons, forests, and even high latitudes, where the number of visible LEO satellites is sparse. We present the algorithm principle, processing flow, and corresponding state equations and observation equations of multi-aircraft cooperative navigation and positioning based on the DWAPC-TSM system.

### 3.1. Algorithm Principle

Unlike a single aircraft, a collaborative aircraft not only has its own absolute position, but also has a relative position with other collaborative members. According to the analysis in Section 2, the accuracy of ranging and time synchronization between aircraft is relatively high, and the accuracy is also very high [30]. For two kinds of related but different precision information, the information with high precision can be used to correct the information with low precision. Therefore, it is reasonable to use the relative position information between aircraft to correct the absolute position information output by the navigation equipment of each aircraft [31]. Accordingly, we consider constructing a new measurement variable that contains all of the relative position information among the cooperative members. The state variables are then estimated by filtering with a UKF.

**Figure 3:**
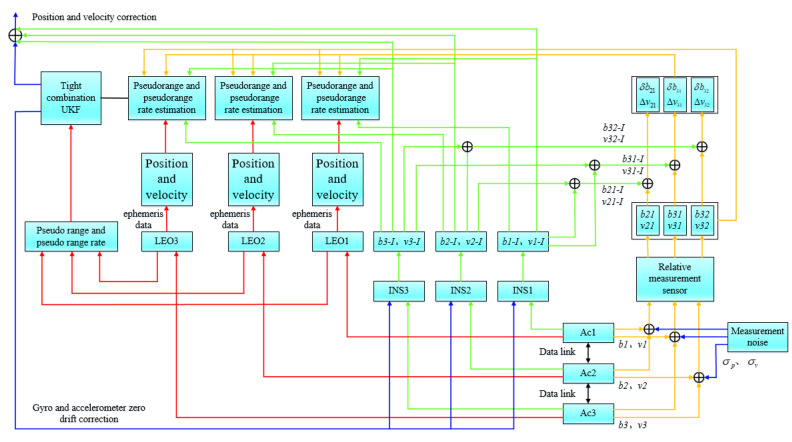
Overall block diagram of the system.

**Figure 4:**
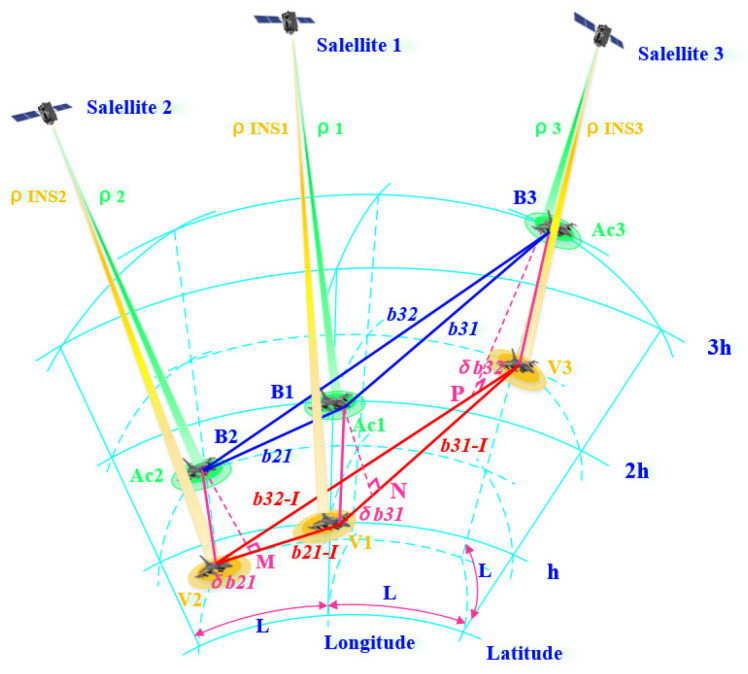
Schematic diagram of collaborative navigation in the DWAPC-TSM system.

Without loss of generality, we only take three aircraft for cooperative navigation as an example. When the formation consists of N (N>3) aircraft, then three aircraft can be combined as a group to form a total of $C_{N}^{3}$ triangles, and finally, the algorithm in this paper is used for collaborative navigation. Assuming that a data link is used for communication between aircraft, inter-aircraft time synchronization is guaranteed according to the DWAPC-TSM system, and the communication delay has been calibrated and corrected. In this way, it can be ensured that information such as the relative distance between two aircraft, the relative velocity, and the output parameters of its own navigation system are transmitted in real time. The overall processing block diagram of the system is shown in Figure 3, and the corresponding collaborative navigation principle diagram is shown in Figure 4.

According to Figure 3, we take three satellites and three aircraft as examples, and name the satellites and aircraft satellite 1, satellite 2, satellite 3, aircraft 1 (Ac1), aircraft 2 (Ac2), and aircraft 3 (Ac3). We assume that the position coordinates of the satellites in the ECEF coordinate system are $\left(x_{si},y_{si},z_{si}\right)$, where i=1,2,3, corresponding to the coordinate positions of the three satellites, respectively. We assume that the real position of the aircraft in the ECEF coordinate system is $\left(x_{uj},y_{uj},z_{uj}\right)$, the corresponding point is $B_{j}$, the position obtained by the INS solution is $\left(x_{Ij},y_{Ij},z_{Ij}\right)$, and the corresponding point is $V_{j}$, j=1,2,3. The distances between aircraft measured by the ranging sensor are *b21* (between Ac2 and Ac1), *b31* (between Ac3 and Ac1), and *b32* (between Ac3 and Ac2). In addition, we assume that the corresponding relative velocities measured by the velocity sensor are *v21*, *v31*, and *v32*. Correspondingly, *b21-I*, *b31-I*, and *b32-I* represent the relative distance between each INS measured by the ranging sensor. *v21-I*, *v31-I*, and *v32-I* are the corresponding relative velocities. $\rho_{i}$ is the pseudorange (*i* = 1, 2, 3) between aircraft *i* and satellite *i* calculated by satellite *i* through the ephemeris, and $\rho_{INSj}$ is the distance between aircraft *j* and satellite *i* calculated by the INS (*j* = 1, 2, 3 and *j = i*). For the convenience of analysis, we set the latitude and longitude distances between aircraft to *L* and the height to *h*. The specific process is as follows:

① First, we select the best satellite in the line of sight of each aircraft through the geometric dilution precision (GDOP) satellite selection algorithm [32], and each aircraft uses the corresponding ephemeris data provided by the selected corresponding LEO satellite to calculate the position and velocity of the corresponding satellite. Then, we calculate the corresponding pseudorange and pseudorange rate observations according to the position provided by the INS combined with the LEO receiver. At the same time, each aircraft obtains the distance measurement value and velocity measurement value between the corresponding aircraft through its own distance measurement and velocity measurement sensor, wherein, through the distance measurement value (baseline measurement rate), the corresponding distance measurement rate is obtained;

② Then, the previously calculated difference in pseudorange and pseudorange rates, and the difference in range and rate, together with velocity and velocity difference (obtainable via the data link), are used as tightly integrated navigation observation values of the system, and the optimal estimated value of the LEO satellite, the relative ranging and velocity sensor, and the INS error are obtained through the unscented Kalman filter (UKF);

③ Finally, the LEO system, the relative ranging and velocity measurement system and the INS are corrected.

In this process, we will discuss two scenarios.

Scenario 1. No Altimeter Assistance Scenario

When not using altimeters:

(16) $$\rho_{i}=\sqrt{{\left(x_{si}-x_{uj}\right)}^{2}+{\left(y_{si}-y_{uj}\right)}^{2}+{\left(z_{si}-z_{uj}\right)}^{2}}\begin{array}{cc} , & \left(i=j=1,2,3\right) \end{array}$$

(17) $$\rho_{INSi}=\sqrt{{\left(x_{si}-x_{Ij}\right)}^{2}+{\left(y_{si}-y_{Ij}\right)}^{2}+{\left(z_{si}-z_{Ij}\right)}^{2}}\begin{array}{cc} , & \left(i=j=1,2,3\right) \end{array}$$

After obtaining the corresponding distance and distance rate observations, the position solution for aircraft *i* (*i* = 1,2,3) can be estimated in combination with the UKF algorithm, and then the state variables are updated to cycle this process. Finally, the cooperative navigation and positioning algorithm is realized.

Scenario 2. Altimeter Assist Scenario

According to reference [33], the navigation and positioning error can be effectively improved with the aid of an altimeter. Therefore, we add the altimeter and regard the center of mass of the Earth as a satellite; then, we obtain:

(18) $$\rho_{Hi}=R_{er}+H_{i}\begin{array}{cc} & \left(i=1,2,3\right) \end{array}$$

(19) $$\rho_{IHi}=\sqrt{{\left(x_{Ii}-0\right)}^{2}+{\left(y_{Ii}-0\right)}^{2}+{\left(z_{Ii}-0\right)}^{2}}\begin{array}{cc} & \left(i=1,2,3\right) \end{array}$$

where $R_{er}$ is the average Earth radius, and $H_{i}$ is the altimeter reading of aircraft *i*. According to Equations (18) and (19), combined with Equations (16) and (17), the subsequent processing flow is consistent with the case without altimeter assistance, and finally, the altimeter-assisted co-navigation and localization algorithm is complete.

### 3.2. Multi-Aircraft Collaborative Navigation Combination Model Based on Tight Combination

#### 3.2.1. State Equation

According to Figure 3 and Figure 4, the state variables of collaborative navigation can be expressed as [34]:

(20) $$x_{SINS}\;=\;{\left[p_{1},p_{2},p_{3},v_{1},v_{2},v_{3}\right]}^{T}$$

where $p_{1}=[p_{1N},p_{1E},p_{1D}]$, $p_{2}=[p_{2N},p_{2E},p_{2D}]$, and $p_{3}=[p_{3N},p_{3E},p_{3D}]$ are the position coordinate vectors of aircrafts 1, 2, and 3 in the Earth-centered Earth-fixed (ECEF) system along the north-east-down (NED) direction, respectively; $v_{1}=[v_{1N},v_{1E},v_{1D}]$, $v_{2}=[v_{2N},v_{2E},v_{2D}]$, and $v_{3}=[v_{3N},v_{3E},v_{3D}]$ are the velocity vectors of aircraft 1, 2, and 3 in the ECEF system along the NED direction, respectively.

Correspondingly, the state equation of the INS is as follows:

(21) $${\dot{x}}_{SINS}(t)=f_{SINS}(t)x_{SINS}(t)+g_{SINS}(t)w_{SINS}(t)$$

where $f_{SINS}(t)$ is a 18 × 18-dimensional state transition matrix; $x_{SINS}(t)$ is a 18 × 1-dimensional state variable (see Equation (20)); $g_{SINS}(t)$ is a 18 × 9-dimensional noise driving matrix; and $w_{SINS}(t)$ is a 9 × 1-dimensional process noise vector. The expressions of $f_{SINS}(t)$ and $g_{SINS}(t)$ can be found in [34], and the expression of $w_{SINS}(t)$ is as follows:

(22) $$w_{SINS}(t)={\left[\sigma_{p1},\sigma_{p2},\sigma_{p3}\right]}^{T}$$

where $\sigma_{p1}={\left[\sigma_{p1N},\sigma_{p1E},\sigma_{p1D}\right]}^{T}$, $\sigma_{p2}={\left[\sigma_{p2N},\sigma_{p2E},\sigma_{p2D}\right]}^{T}$, $\sigma_{p3}={\left[\sigma_{p3N},\sigma_{p3E},\sigma_{p3D}\right]}^{T}$.

According to Equations (20)–(22), the state equation of the combined system can be obtained as follows:

(23) $$\dot{x}(t)=f(t)x(t)+g(t)w(t)$$

where $f=f_{INS}$, $x=x_{INS}$, $g=g_{INS}$, and $w=w_{INS}$.

#### 3.2.2. Observation Equation

According to Figure 4, the relationship between the measured variable and the state variable is as follows:

(24) $$\left\{ \begin{array}{l} b_{21}=\sqrt{{\left(x_{2}-x_{1}\right)}^{2}+{\left(y_{2}-y_{1}\right)}^{2}+{\left(z_{2}-z_{1}\right)}^{2}}+\sigma_{b21}=b_{21}^{r}+\sigma_{b21}\\ b_{31}=\sqrt{{\left(x_{3}-x_{1}\right)}^{2}+{\left(y_{3}-y_{1}\right)}^{2}+{\left(z_{3}-z_{1}\right)}^{2}}+\sigma_{b31}=b_{31}^{r}+\sigma_{b31}\\ b_{32}=\sqrt{{\left(x_{3}-x_{2}\right)}^{2}+{\left(y_{3}-y_{2}\right)}^{2}+{\left(z_{3}-z_{2}\right)}^{2}}+\sigma_{b32}=b_{32}^{r}+\sigma_{b32} \end{array}\right.$$

(25) $$\left\{ \begin{array}{l} b_{21-I}=\sqrt{{\left(x_{I2}-x_{I1}\right)}^{2}+{\left(y_{I2}-y_{I1}\right)}^{2}+{\left(z_{I2}-z_{I1}\right)}^{2}}\\ b_{31-I}=\sqrt{{\left(x_{I3}-x_{I1}\right)}^{2}+{\left(y_{I3}-y_{I1}\right)}^{2}+{\left(z_{I3}-z_{I1}\right)}^{2}}\\ b_{32-I}=\sqrt{{\left(x_{I3}-x_{I2}\right)}^{2}+{\left(y_{I3}-y_{I2}\right)}^{2}+{\left(z_{I3}-z_{I2}\right)}^{2}} \end{array}\right.$$

where $\sigma_{b21}$, $\sigma_{b31}$, and $\sigma_{b32}$ are the corresponding ranging noise.

A Taylor expansion of Equation (25) is obtained at its true values $\left(x_{i},y_{i},z_{i}\right)$, with *i* = 1, 2, 3, and by ignoring the second-order and higher-order terms:

(26) $$\left\{ \begin{array}{l} b_{21-I}=b_{21}^{r}+\frac{\left(x_{2}-x_{1})-(x_{s2}-x_{s1}\right)}{b_{21}^{r}}\Delta (x_{2}-x_{1})+\frac{\left(y_{2}-y_{1})-(y_{s2}-y_{s1}\right)}{b_{21}^{r}}\Delta (y_{2}-y_{1})\\ \begin{array}{ccc} & & +\frac{\left(z_{2}-z_{1})-(z_{s2}-z_{s1}\right)}{b_{21}^{r}}\Delta (z_{2}-z_{1}) \end{array}\\ b_{31-I}=b_{31}^{r}+\frac{\left(x_{3}-x_{1})-(x_{s3}-x_{s1}\right)}{b_{31}^{r}}\Delta (x_{3}-x_{1})+\frac{\left(y_{3}-y_{1})-(y_{s3}-y_{s1}\right)}{b_{31}^{r}}\Delta (y_{3}-y_{1})\\ \begin{array}{ccc} & & +\frac{\left(z_{3}-z_{1})-(z_{s3}-z_{s1}\right)}{b_{31}^{r}}\Delta (z_{3}-z_{1}) \end{array}\\ b_{32-I}=b_{32}^{r}+\frac{\left(x_{3}-x_{2})-(x_{s3}-x_{s2}\right)}{b_{32}^{r}}\Delta (x_{3}-x_{2})+\frac{\left(y_{3}-y_{2})-(y_{s3}-y_{s2}\right)}{b_{32}^{r}}\Delta (y_{3}-y_{2})\\ \begin{array}{ccc} & & +\frac{\left(z_{3}-z_{2})-(z_{s3}-z_{s2}\right)}{b_{32}^{r}}\Delta (z_{3}-z_{2}) \end{array} \end{array}\right.$$

where $b_{21}^{r}$, $b_{31}^{r}$, and $b_{32}^{r}$ represent the real distances between the subscripts of the two aircraft.

Let:

$$\begin{array}{l} \varepsilon_{21x}=\frac{\left(x_{2}-x_{1})-(x_{s2}-x_{s1}\right)}{b_{21}^{r}},\;\varepsilon_{21y}=\frac{\left(y_{2}-y_{1})-(y_{s2}-y_{s1}\right)}{b_{21}^{r}},\;\varepsilon_{21z}=\frac{\left(z_{2}-z_{1})-(z_{s2}-z_{s1}\right)}{b_{21}^{r}};\\ \varepsilon_{31x}=\frac{\left(x_{3}-x_{1})-(x_{s3}-x_{s1}\right)}{b_{31}^{r}},\;\varepsilon_{31y}=\frac{\left(y_{3}-y_{1})-(y_{s3}-y_{s1}\right)}{b_{31}^{r}},\;\varepsilon_{31z}=\frac{\left(z_{3}-z_{1})-(z_{s3}-z_{s1}\right)}{b_{31}^{r}};\\ \varepsilon_{32x}=\frac{\left(x_{3}-x_{2})-(x_{s3}-x_{s2}\right)}{b_{32}^{r}},\;\varepsilon_{32y}=\frac{\left(y_{3}-y_{2})-(y_{s3}-y_{s2}\right)}{b_{32}^{r}},\varepsilon_{32z}=\frac{\left(z_{3}-z_{2})-(z_{s3}-z_{s2}\right)}{b_{32}^{r}} \end{array}$$

Then, Equation (26) can be simplified as follows:

(27) $$\left\{ \begin{array}{l} b_{21-I}=b_{21}^{r}+\varepsilon_{21x}\Delta (x_{2}-x_{1})+\varepsilon_{21y}\Delta (y_{2}-y_{1})+\varepsilon_{21z}\Delta (z_{2}-z_{1})\\ b_{31-I}=b_{31}^{r}+\varepsilon_{31x}\Delta (x_{3}-x_{1})+\varepsilon_{31y}\Delta (y_{3}-y_{1})+\varepsilon_{31z}\Delta (z_{3}-z_{1})\\ b_{32-I}=b_{32}^{r}+\varepsilon_{32x}\Delta (x_{3}-x_{2})+\varepsilon_{32y}\Delta (y_{3}-y_{2})+\varepsilon_{32z}\Delta (z_{3}-z_{2}) \end{array}\right.$$

From Equations (24) and (27), the ranging observation equations of the INS and LEO can be obtained as follows:

(28) $$\left\{ \begin{array}{l} \delta b_{21}=b_{21}-b_{21-I}=\varepsilon_{21x}\Delta (x_{2}-x_{1})+\varepsilon_{21y}\Delta (y_{2}-y_{1})+\varepsilon_{21z}\Delta (z_{2}-z_{1})+\sigma_{b21}\\ \delta b_{31}=b_{31}-b_{31-I}=\varepsilon_{31x}\Delta (x_{3}-x_{1})+\varepsilon_{31y}\Delta (y_{3}-y_{1})+\varepsilon_{31z}\Delta (z_{3}-z_{1})+\sigma_{b31}\\ \delta b_{32}=b_{32}-b_{32-I}=\varepsilon_{32x}\Delta (x_{3}-x_{2})+\varepsilon_{32y}\Delta (y_{3}-y_{2})+\varepsilon_{32z}\Delta (z_{3}-z_{2})+\sigma_{b32} \end{array}\right.$$

written in matrix form:

(29) $$z_{b}=h_{b}x+m_{b}$$

where $h_{b}=[O_{3n\times 6}h_{b1}O_{3n\times 6}h_{b2}]$, *n* is the number of satellites; $h_{b1}=\left[\begin{array}{ccc} \varepsilon_{21x} & \varepsilon_{21y} & \varepsilon_{21z}\\ \varepsilon_{31x} & \varepsilon_{31y} & \varepsilon_{31z}\\ \varepsilon_{32x} & \varepsilon_{32y} & \varepsilon_{32z} \end{array}\right]\nabla C_{n}^{e}$, $h_{b2}=\left[\begin{array}{c} -1\\ -1\\ -1 \end{array}\begin{array}{c} 0\\ 0\\ 0 \end{array}\right]$, $\nabla C_{n}^{e}$ is the state transition matrix from the error of the geographic system to the error of the ECEF system [35], and $m_{b}=[\sigma_{b21},\sigma_{b31},\sigma_{b32}{]}^{T}$.

In the same way, the ranging rate of the INS and LEO can be obtained:

(30) $$\left\{ \begin{array}{l} {\dot{b}}_{21-I}={\dot{b}}_{21}^{r}+{\dot{\varepsilon }}_{21x}\Delta ({\dot{x}}_{2}-{\dot{x}}_{1})+{\dot{\varepsilon }}_{21y}\Delta ({\dot{y}}_{2}-{\dot{y}}_{1})+{\dot{\varepsilon }}_{21z}\Delta ({\dot{z}}_{2}-{\dot{z}}_{1})\\ {\dot{b}}_{31-I}={\dot{b}}_{31}^{r}+{\dot{\varepsilon }}_{31x}\Delta ({\dot{x}}_{3}-{\dot{x}}_{1})+{\dot{\varepsilon }}_{31y}\Delta ({\dot{y}}_{3}-{\dot{y}}_{1})+{\dot{\varepsilon }}_{31z}\Delta ({\dot{z}}_{3}-{\dot{z}}_{1})\\ {\dot{b}}_{32-I}={\dot{b}}_{32}^{r}+{\dot{\varepsilon }}_{32x}\Delta ({\dot{x}}_{3}-{\dot{x}}_{2})+{\dot{\varepsilon }}_{32y}\Delta ({\dot{y}}_{3}-{\dot{y}}_{2})+{\dot{\varepsilon }}_{32z}\Delta ({\dot{z}}_{3}-{\dot{z}}_{2}) \end{array}\right.$$

By taking the derivative of Equation (24), determining its difference from Equation (30), and omitting the tedious derivation, the observation equation based on the ranging rate can be obtained:

(31) $$\left\{ \begin{array}{l} \delta {\dot{b}}_{21}={\dot{b}}_{21}-{\dot{b}}_{21-I}={\dot{\varepsilon }}_{21x}\Delta ({\dot{x}}_{2}-{\dot{x}}_{1})+{\dot{\varepsilon }}_{21y}\Delta ({\dot{y}}_{2}-{\dot{y}}_{1})+{\dot{\varepsilon }}_{21z}\Delta ({\dot{z}}_{2}-{\dot{z}}_{1})+{\dot{\sigma }}_{b21}\\ \delta {\dot{b}}_{31}={\dot{b}}_{31}-{\dot{b}}_{31-I}={\dot{\varepsilon }}_{31x}\Delta ({\dot{x}}_{3}-{\dot{x}}_{1})+{\dot{\varepsilon }}_{31y}\Delta ({\dot{y}}_{3}-{\dot{y}}_{1})+{\dot{\varepsilon }}_{31z}\Delta ({\dot{z}}_{3}-{\dot{z}}_{1})+{\dot{\sigma }}_{b31}\\ \delta {\dot{b}}_{32}={\dot{b}}_{32}-{\dot{b}}_{32-I}={\dot{\varepsilon }}_{32x}\Delta ({\dot{x}}_{3}-{\dot{x}}_{2})+{\dot{\varepsilon }}_{32y}\Delta ({\dot{y}}_{3}-{\dot{y}}_{2})+{\dot{\varepsilon }}_{32z}\Delta ({\dot{z}}_{3}-{\dot{z}}_{2})+{\dot{\sigma }}_{b32} \end{array}\right.$$

which is written in matrix form as:

(32) $$z_{\dot{b}}=h_{\dot{b}}x+m_{\dot{b}}$$

where $h_{\dot{b}}=[O_{3n\times 3}h_{\dot{b}1}O_{3n\times 9}h_{\dot{b}2}]$, $h_{\dot{b}1}=\left[\begin{array}{ccc} \varepsilon_{21x} & \varepsilon_{21y} & \varepsilon_{21z}\\ \varepsilon_{31x} & \varepsilon_{31y} & \varepsilon_{31z}\\ \varepsilon_{32x} & \varepsilon_{32y} & \varepsilon_{32z} \end{array}\right]C_{n}^{e}$, $h_{\dot{b}2}=\left[\begin{array}{c} -1\\ -1\\ -1 \end{array}\begin{array}{c} 0\\ 0\\ 0 \end{array}\right]$, $m_{\dot{b}}=[{\dot{\sigma }}_{b21},{\dot{\sigma }}_{b31},{\dot{\sigma }}_{b32}]$, and $C_{n}^{e}$ is the coordinate transformation matrix [36].

The velocity measurement can be obtained through the velocity sensor, and the corresponding relative velocity error observation is:

(33) $$\left\{ \begin{array}{l} \Delta v_{21}=v_{21}-v_{21-I}+\sigma_{v21}\\ \Delta v_{31}=v_{31}-v_{31-I}+\sigma_{v321}\\ \Delta v_{32}=v_{32}-v_{32-I}+\sigma_{v32} \end{array}\right.$$

According to reference [16], we supplement the corresponding pseudoranges to obtain the final observation equation of the multi-aircraft cooperative navigation tightly integrated navigation algorithm of the DWAPC-TSM system:

(34) $$Z(t)=H(t)X(t)+M(t)=\left[\begin{array}{l} z_{b}(t)\\ z_{\dot{b}}(t)\\ z_{v}(t)\\ z_{\rho }(t)\\ z_{\dot{\rho }}(t) \end{array}\right]=\left[\begin{array}{l} h_{b}(t)x(t)\\ h_{\dot{b}}(t)x(t)\\ h_{v}(t)x(t)\\ h_{\rho }(t)x(t)\\ h_{\dot{\rho }}(t)x(t) \end{array}\right]+\left[\begin{array}{l} m_{b}(t)\\ m_{\dot{b}}(t)\\ m_{v}(t)\\ m_{\rho }(t)\\ m_{\dot{\rho }}(t) \end{array}\right]$$

where $h_{v}=[v_{21}-v_{21-I},v_{31}-v_{31-I},v_{32}-v_{32-I}]$ and $m_{v}=[\sigma_{v21},\sigma_{v31},\sigma_{v32}]$. The details of $h_{\rho }(t)$, $h_{\dot{\rho }}(t)$, $m_{\rho }(t)$, and $m_{\dot{\rho }}(t)$ can be found in [16].

### 3.3. Other Models

(1) Influence of aircraft spacing range on satellite observability

Whether a satellite signal can be received mainly depends on the following factors [37]:

① Whether the Earth has affected the propagation of the LEO satellite signal

**Figure 5:**
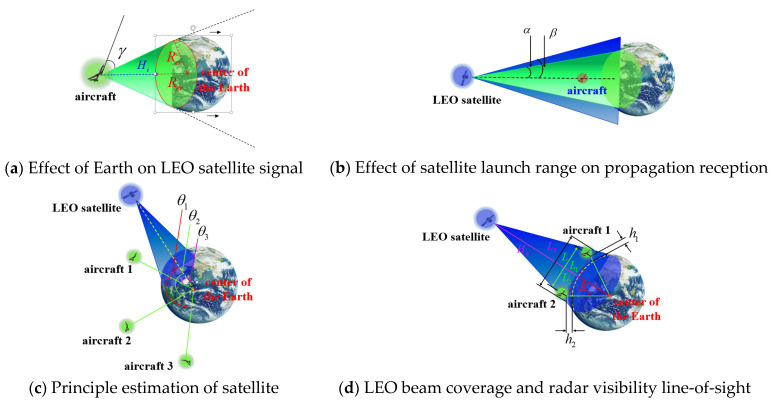
Schematic diagram of the effect of aircraft spacing range on satellite observability.

As shown in Figure 5a, if the satellite is located in the part indicated by the black dotted line and the black arrow in Figure 5a, the aircraft in the figure cannot receive the signal of the satellite, that is, the satellite signals from satellites are invisible.

② Whether the LEO receiver is located within the range of the LEO satellite transmitting antenna.

As shown in Figure 5b, this situation is mainly aimed at the LEO receiver on the aircraft. The launch angle of the LEO satellite signal is around β ($\beta =21.3^{o}$ for the medium earth orbit (MEO) satellite, but for the LEO satellite, since its orbit is lower than that of the MEO satellite, the value of β is larger), which is larger than the satellite-to-horizontal angle α ($\alpha =13.9^{o}$, for the same reason, for LEO satellites, the value of α is larger), as long as β>α is guaranteed, it can ensure that some aircraft with higher flying altitudes can receive more information at high altitudes signals from LEO satellites. However, for those aircraft beyond the launch angle, the signal will not be received.

③ Using the opening angle θ between the satellite-geocenter-aircraft, it can be estimated whether the satellite signal can be received or not.

As shown in Figure 5c, using the opening angle θ formed by the satellite-geocenter-aircraft, it can be estimated whether the satellite is blocked by the Earth’s shadow,

When $\theta =\theta_{1}$, θ is an obtuse angle at this time, so the two are blocked by the Earth and cannot receive the signal;

When $\theta =\theta_{2}$, θ is a right angle at this time, which is a critical situation, and it is generally considered that satellite signal cannot be received;

When $\theta =\theta_{3}$, θ is an acute angle at this time, and the satellite signal can be received. According to the basic knowledge of geometry, the opening angle formed by the satellite-geocenter-user can be calculated by the law of cosines.

④ Other factors

Other factors, such as the receiving angle of the LEO receiver and the signal-to-noise ratio (SNR) if it is high enough, also affect whether the navigation signal of a certain satellite can be received.

In view of the needs of the task of formation aircraft, the usual formation aircraft are also equipped with line-of-sight radar or over-the-horizon radar. Therefore, based on actual needs, we combine the LEO satellite beam coverage and the radar line-of-sight formula to give a range of aircraft formation spacing suitable for practical applications, according to Figure 5d:

(35) $$\left\{ \begin{array}{l} L_{1}+L_{2}=L\\ L_{1}^{2}+R_{er}^{2}=(R_{er}^{}+h_{1}{)}^{2}\\ L_{2}^{2}+R_{er}^{2}=(R_{er}^{}+h_{2}{)}^{2} \end{array}\right.$$

Solving the equation, we can obtain:

(36) $$L=4.12(\sqrt{h_{1}}+\sqrt{h_{2}})$$

In addition, the LEO satellite orbit height is usually in the range of 500 km~1500 km [38]. For the sake of conservativeness, we use the satellite’s opening angle α to the horizontal plane to calculate, and take $\alpha =13.9^{o}$, and the satellite orbital height is $H_{S}$ = 500 km. For most aircraft, the flight altitude is generally within the stratosphere or troposphere [39], within 30 km. Here, we take the heights of aircraft 1 and 2 as: $h_{1}=h_{2}=30$ km, then $L_{S}=H_{S}\tan13.9^{o}\approx 123.74$ km, $L=4.12(\sqrt{h_{1}}+\sqrt{h_{2}})\approx 1.43$ km, and for aircraft 3, on the premise that the LEO signal can be received, in theory, it can be located at any position in a cone with $L_{S}$ as the bottom diameter and 2α as the top angle, but considering the actual situation such as formation coordination, in practice, we can consider it to be located anywhere on a sphere of radius $L_{S}$. As long as it is within this range, the distance between the three aircraft has no effect on the observability of the satellite. Of course, the distance between the aircraft cannot be too small, and sufficient safety coordination and braking distance should be preserved. We also set the simulation parameters based on this in the subsequent simulation verification. For other opening angles α that are greater than the satellite to the horizontal plane, it can be deduced from the properties of the tan function that in the $\left[0,90^{o}\right)$ interval, its value increases with the increase of α.

(2) Satellite selection algorithm and other models

For other models involved in the article, such as the atmospheric drag model, Earth aspherical perturbation model, ionosphere and troposphere model, and multipath and noise interference model, refer to references [32,40,41,42,43]; these methods were adopted in the algorithm, or we learned from these models or ideas. Due to space limitations, we will not elaborate on these models here.

## 4. Simulation Verification and Analysis

Regarding constellation selection, we use the Kuiper constellation [44] for modeling and simulation, and the flight maneuver of the aircraft includes take-off, uniform linear motion, steering, and climbing. The aircraft and constellation parameters are shown in Table 1, and the main parameters of the INS and relative ranging and velocity sensors are shown in Table 2. Here, we believe that the key performance indicators of INS equipment equipped in different aircraft are highly consistent.

According to Table 1 and Table 2 and the abovementioned related theories, we carried out simulation experiments according to the two scenarios in Section 3.1. Some abbreviation involved in the experiments are as follows:

− NPE: north position error;
− EPE: east position error;
− DPE: down position error;
− NVE: north speed error;
− EVE: east velocity error;
− DVE: down velocity error;
− Ac*i* INS indicates the INS equipment used by the *i*-th aircraft;
− Ac*i*|Alt = H m indicates that aircraft *i* is H m in the altimeter algorithm performance below, where *i* = 1,2,3, represents the *i*-th aircraft, Alt represents the altimeter, and H = 0 m, 5 m, and 15 m.

### 4.1. Multi-Aircraft Cooperative Navigation and Positioning Algorithm without Altimeter Assistance

**Figure 6:**
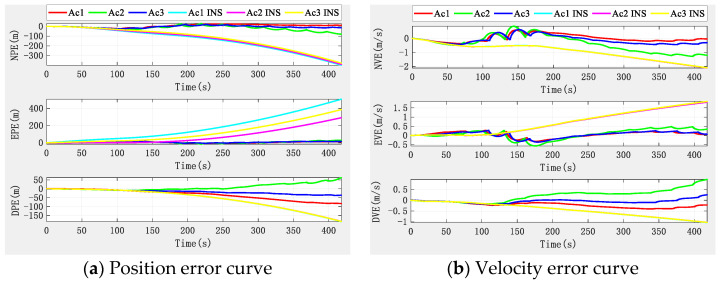

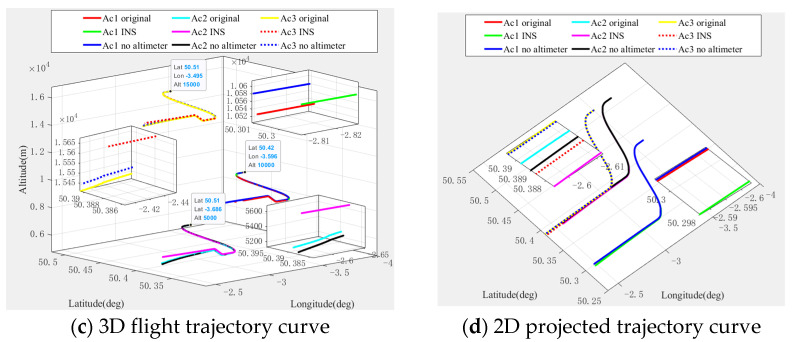
Navigation and positioning error curve of multi-aircraft cooperative navigation in the DWAPC-TSM system without altimeter assistance.

The simulation results, according to the algorithm principle and setting conditions, without the aid of the altimeter, are shown in Figure 6, in which, as a comparison, we added the positioning results of pure INS collaborative navigation.

**Figure 7:**
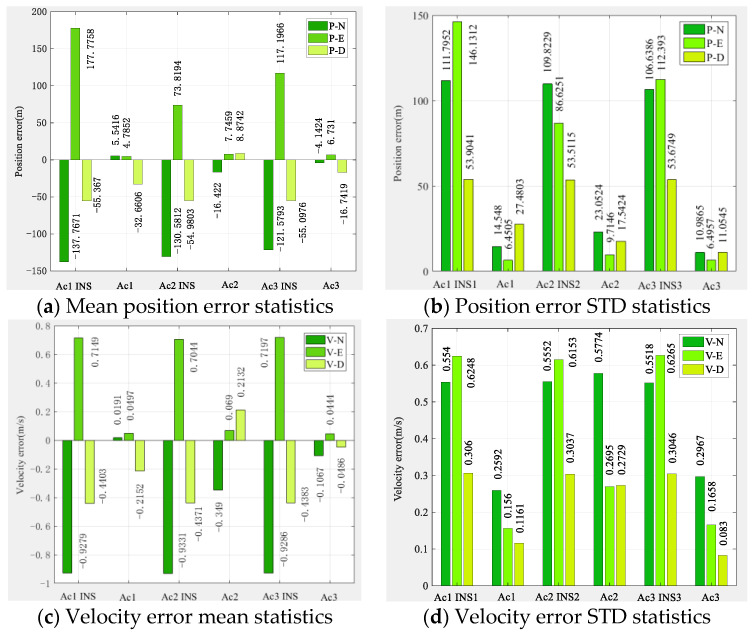
Statistical histogram of the multi-aircraft cooperative navigation and positioning indicators of the DWAPC-TSM system without the aid of an altimeter.

Figure 6 shows that the altimeter-free multi-aircraft cooperative navigation and positioning algorithm using the DWAPC-TSM system without altimeter assistance can effectively suppress INS divergence, and its performance is also better than the pure INS cooperative navigation and positioning performance. Figure 6 also shows that, due to the dead reckoning principle upon which INS relies, its navigation and positioning error curve does not fluctuate much, and appears to be very flat, but the error is in a divergent state. However, the curve of the multi-aircraft cooperative navigation and positioning algorithm using the DWAPC-TSM system exhibits certain fluctuations, except for the down position error and velocity error, and the final error curve converges to 0. From the final three-dimensional trajectory curve and projection curve, compared with the pure INS collaborative navigation algorithm, each aircraft can improve the performance of the collaborative navigation and positioning algorithm, as well as suppress the divergence of the INS. To qualitatively compare with the pure INS cooperative navigation and positioning algorithm, the statistical results of the navigation and positioning indicators are shown in Figure 7.

As Figure 7 shows, the position error of pure INS cooperative navigation is significantly higher than that of the DWAPC-TSM system multi-aircraft cooperative navigation and positioning algorithm, regardless of the mean and standard deviation, and its maximum positioning accuracies are approximately 146.1312 m (Ac1 INS), 109.8229 m (Ac2 INS), and 112.3930 m (Ac3 INS); the corresponding maximum velocity accuracy is 0.62 m/s (Ac1 INS), 0.61 m/s (Ac2 INS), and 0.62 m/s (Ac3 INS). However, for the altimeter-assisted DWAPC-TSM system multi-aircraft cooperative navigation and positioning algorithm, the corresponding maximum positioning accuracies are 27.4803 m (Ac1), 23.2524 m (Ac2), and 11.0545 m (Ac3), and the corresponding maximum velocity accuracies are 0.2592 m/s, 0.5774 m/s, and 0.2967 m/s. In comparison, the performance of the multi-aircraft cooperative navigation and positioning algorithm based on the WP-CTM system without altimeter assistance is greatly improved, as compared with that of the pure INS cooperative positioning algorithm, which can meet the basic location service requirements in challenging environments.

### 4.2. Altimeter-Assisted Multi-Aircraft Cooperative Navigation and Positioning Algorithm

**Figure 8:**
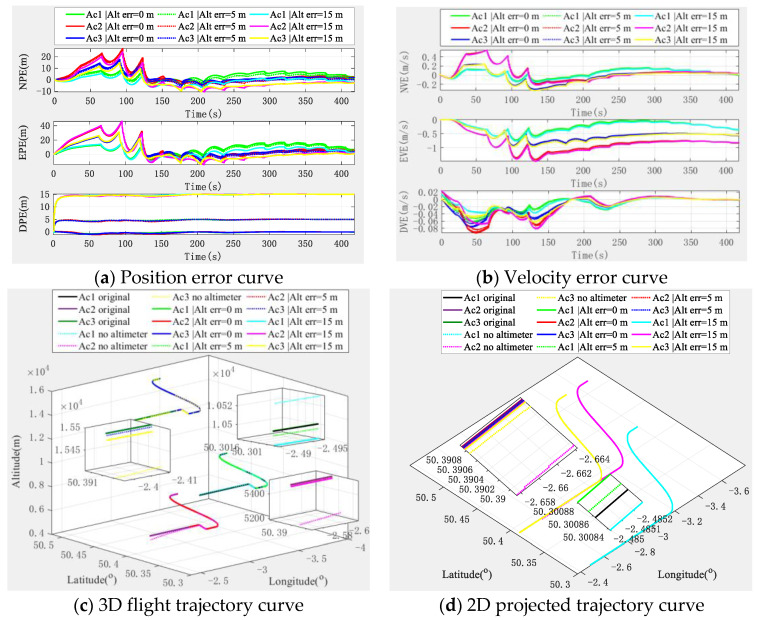
Navigation and positioning error curve of multi-aircraft cooperative navigation in the DWAPC-TSM system with the aid of an altimeter.

With the aid of an altimeter, the simulation experiment results of the cooperative navigation and positioning algorithm based on the DWAPC-TSM system are shown in Figure 8.

**Figure 9:**
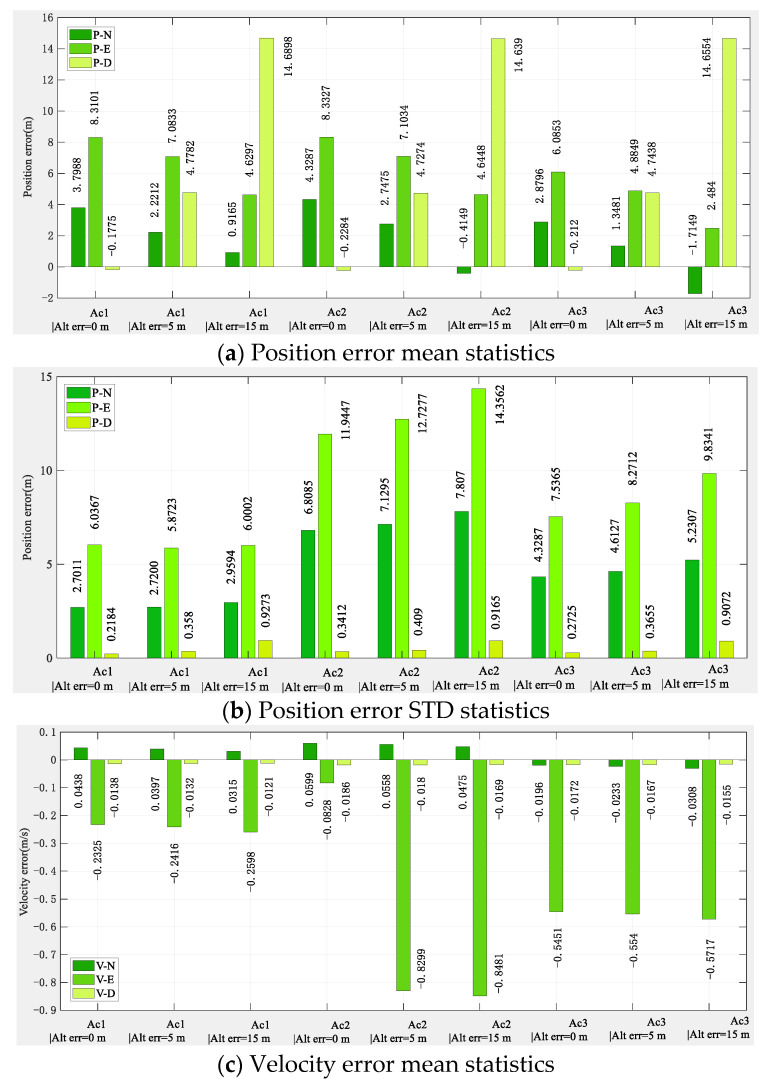

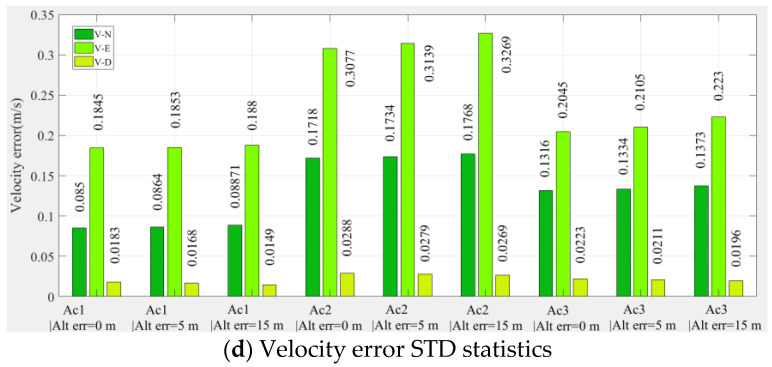
Statistical histogram of the multi-aircraft cooperative navigation and positioning index of the DWAPC-TSM system assisted by an altimeter.

Figure 8 shows that an altimeter greatly improves the down position error and velocity error of the multi-aircraft cooperative navigation and positioning curve of the DWAPC-TSM system, and the position error and velocity error in other directions are significantly improved. In addition, for the same aircraft, with the increase in the altimeter error, the corresponding positioning error also increases, which also shows that the accuracy and cost level of the altimeter should be considered for practical engineering applications. In addition, the final cooperative and positioning trajectory curve shows that with the assistance of altimeters with different deviations, the cooperative and positioning performance of the algorithm can be further improved, even when the altimeter deviation is 15 m. Compared with the cooperative navigation and positioning algorithm without altimeter assistance, this system also has a better performance. Similarly, we categorized the navigation and positioning error indicators of the multi-aircraft cooperative navigation and positioning algorithm of the DWAPC-TSM system with different altimeters, as shown in Figure 9.

According to the statistical results and in Figure 9:

(1) When the altimeter has no deviation, the maximum position error accuracies of the aircraft are 6.0367 m (Ac1), 11.9447 m (Ac2), and 7.5365 m (Ac3), and the minimum error accuracies are 0.2184 m (Ac1), 0.3412 m (Ac2), and 0.2725 m (Ac3). Accordingly, the maximum velocity error accuracies of the aircraft are 0.1845 m/s (Ac1), 0.3077 m/s (Ac2), and 0.2045 m/s (Ac3), and the minimum error accuracies are 0.0183 m/s (Ac1), 0.0288 m/s (Ac2), and 0.0196 m/s (Ac3).
(2) In the case of an altimeter error of 5 m, the maximum position error accuracies of the aircraft are 5.8723 m (Ac1), 12.7277 m (Ac2), and 8.2712 m (Ac3), and the minimum error accuracies are 0.3580 m (Ac1), 0.4090 m (Ac2) and 0.3655 m (Ac3). Accordingly, the maximum velocity error accuracies of the aircraft are 0.1853 m/s (Ac1), 0.3139 m/s (Ac2), and 0.2105 m/s (Ac3), and the minimum error accuracies are 0.0168 m/s (Ac1), 0.0279 m/s (Ac2), and 0.0211 m/s (Ac3).
(3) In the case an of altimeter error of 15 m, the maximum position error accuracies of the aircraft are 6.0002 m (Ac1), 14.3562 m (Ac2), and 9.8341 m (Ac3), and the minimum error accuracies are 0.9273 m (Ac1), 0.9165 m (Ac2) and 0.9072 m (Ac3). Accordingly, the maximum velocity error accuracies of the aircraft are 0.1880 m/s (Ac1), 0.3269 m/s (Ac2), and 0.2230 m/s (Ac3), and the minimum error accuracies are 0.0149 m/s (Ac1), 0.0269 m/s (Ac2), and 0.0196 m/s (Ac3).

The comparison of the above altimeter errors shows that, although there are fluctuations in individual index errors, which are mainly caused by the subtle differences between the position of the aircraft and the form of maneuver, the final errors show a downwards trend in navigation and positioning performance with an increase in altimeter error, which is in line with the expectation.

### 4.3. Influence of Aircraft Configuration on Aircraft Positioning Accuracy

The aircraft types considered in Section 4.1 and Section 4.2 are actually all three aircraft located at different positions in the three-dimensional space, as shown in Figure 4. However, in the actual formation flight process, there are still three aircraft that are in collinear (including horizontal collinear and vertical collinear) and coplanar situations, but the coplanar case is basically similar to the situation in Section 4.1 and Section 4.2. Therefore, this subsection, we focus on the simulation analysis of the collinear situation. In addition, according to the analysis in Section 4.1 and Section 4.2, adding an altimeter can effectively improve the navigation and positioning accuracy. Therefore, due to space limitations, we only analyze the unbiased altimeter-assisted situation here.

(1) Horizontal collinear situation

**Figure 10:**
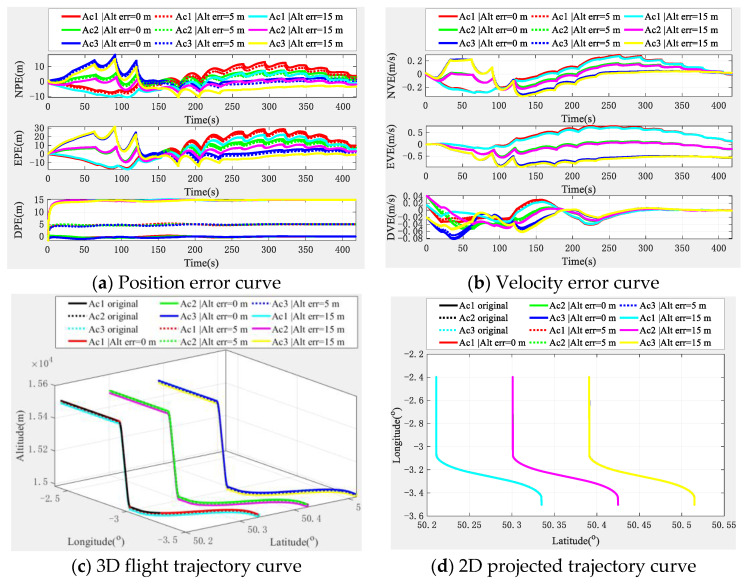
Navigation and positioning error curve when the formation is horizontally collinear.

Strictly speaking, there are two situations of horizontal collinear. The first situation is that the formation is lined up and flying in parallel, and the other situation is that the formation is flying in one column. However, regardless of the situation, the essence is the same, so we only discuss the first case here, and the simulation results are shown in Figure 10.

From Figure 10, we can see that when the formation is flying horizontally and collinearly, the position and velocity error of each aircraft do not diverge, but converge, and with the increase of the altimeter error, there is also a corresponding increase in the navigation and positioning error. The final three-dimensional error and trajectory curve and its projection error trajectory curve also have similar conclusions. It can be seen that the formation horizontal collinear configuration has no effect on the performance of the algorithm.

(2) Vertical collinear situation

**Figure 11:**
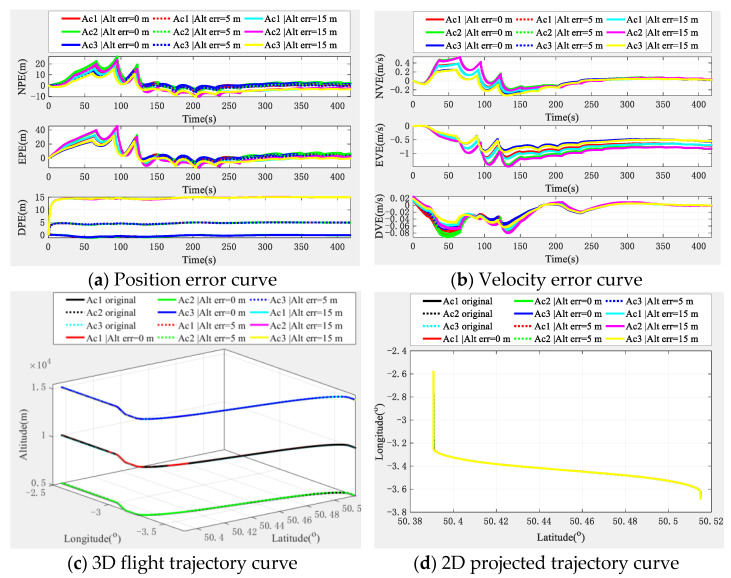
Navigation and positioning error curve when the formation is vertically collinear.

For the vertical collinear flight configuration of the formation, its essence is slightly different from the horizontal collinear situation. The main reason is that the satellite beam coverage is essentially the same for the formation members. The simulation results are shown in Figure 11.

From Figure 11, we can see that in the vertical formation configuration, the navigation and positioning performance of the formation members tends to be consistent, which is also expected; in addition, the navigation and positioning errors of the formation members also converge. Similarly, although the corresponding error increases with the increase of the altimeter error, however, the navigation and positioning errors also show a trend of convergence, which also shows that the navigation and positioning performance of the formation is not affected by it when flying in a vertical configuration.

According to the analysis results of the two situations of horizontal collinear and vertical collinear, it can be demonstrated that even if the formation is in the collinear flight configuration, the navigation and positioning performance is not affected, and in the case of vertical collinearity, the navigation and positioning performance of the formation members tends to be consistent. Therefore, the multi-aircraft cooperative navigation and positioning algorithm based on the TWP-CTM system is generally applicable.

### 4.4. Influence of Relative Positioning Error on Aircraft Positioning Accuracy

In view of the fact that different ranging and velocity sensors may have different measurement accuracy in practice, which will affect the relative positioning accuracy and may affect the final navigation and positioning accuracy, in this section, we focus on the simulation analysis of the influence of different relative positioning accuracy on the final navigation positioning error. Similarly, due to space limitations, we only analyze the unbiased altimeter-assisted situation. Without loss of generality, we only take aircraft 1 as the analysis object.

**Figure 12:**
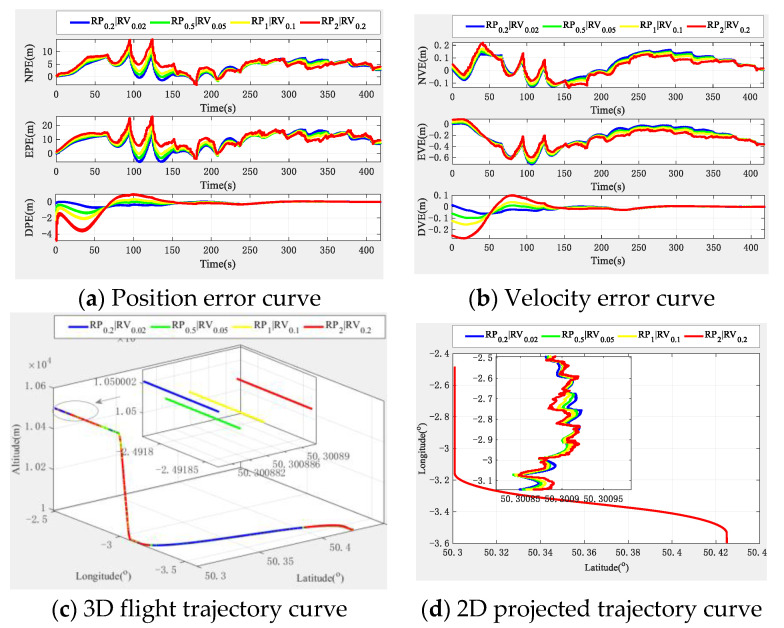
Navigation positioning error curves under different relative measurement accuracy.

According to the current relative ranging and velocity measurement accuracy indicators [45,46], we set the relative position (RP) measurement accuracy as 0.2 m (the case in Section 4.2), 0.5 m, 1 m, and 2 m, respectively; the relative velocity (RV) measurement accuracy as 0.02 $m\times s^{-1}$ (the case in Section 4.2), 0.05 $m\times s^{-1}$, 0.1 $m\times s^{-1}$, and 0.2 $m\times s^{-1}$ to explore the effect of different relative measurement accuracy on the performance of the algorithm. The simulation results are shown in Figure 12.

It can be seen from the simulation results in Figure 12 that with the decrease of relative measurement accuracy, the corresponding navigation and positioning performance also gradually deteriorates. However, in general, only the local flight time has a greater impact, but the final navigation and positioning results are convergent. This shows that the multi-aircraft cooperative navigation and positioning algorithm based on the TWP-CTM system is more robust, and can be suitable for flight business requirements equipped with sensors with different measurement accuracy.

## 5. Algorithm Comparison

### 5.1. Comparison of Different LEO Constellations

**Figure 13:**
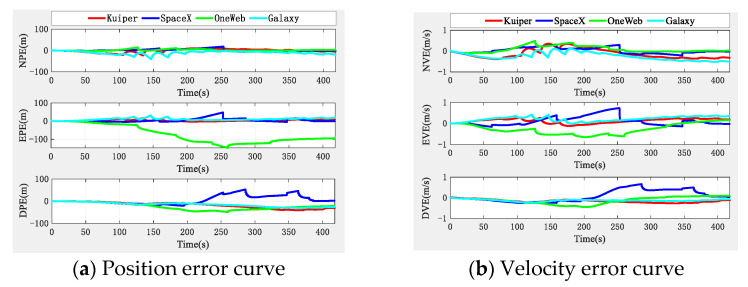

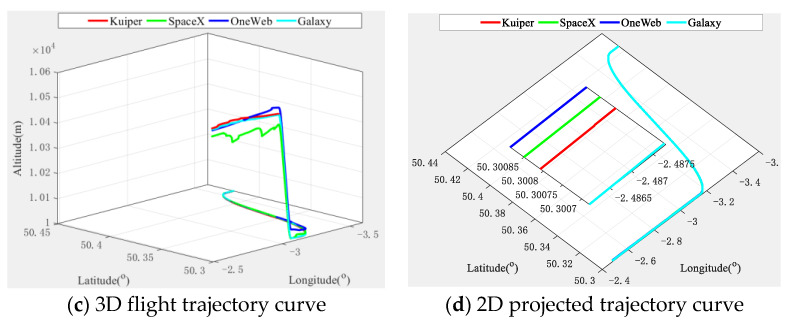
Comparison of altimeter-free multi-aircraft cooperative navigation and positioning algorithms based on the DWAPC-TSM system for different LEO constellations.

**Figure 14:**
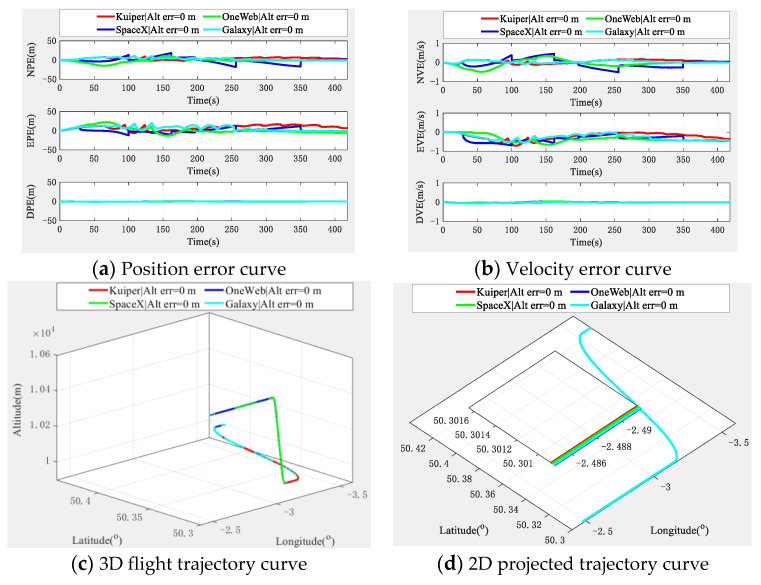
Comparison curve of altimeter-assisted multi-aircraft cooperative navigation and positioning algorithms based on the DWAPC-TSM system for different LEO constellations.

To verify the universality of the multi-aircraft cooperative navigation and positioning algorithm based on the DWAPC-TSM system for the LEO constellation, in this section we compare the current LEO constellations with other types. For the constellation parameters of SpaceX and Oneweb, refer to reference [12]. For the constellation parameters of Galaxy (China), please refer to [47]; these parameters are not listed in this paper. We simulate the single-satellite cooperative navigation and positioning algorithm with and without altimeter assistance. The final results are shown in Figure 13 and Figure 14.

Figure 13 and Figure 14 show that regardless of altimeter assistance, due to the different parameters of each constellation, such as the inclination angle and orbit height and the distribution of satellite orbits which lead to the GDOP value and various interferences of the system, the error curve of the multi-aircraft cooperative navigation and positioning algorithm based on the DWAPC-TSM system varies slightly in individual directions. However, in general, the navigation and positioning performances for each LEO constellation are roughly the same, and with the aid of an altimeter, the performance of the algorithm is significantly improved. From the final three-dimensional trajectory curve and its corresponding projection curve, there is almost no difference between the trajectories of the LEO systems, which means that with the aid of an altimeter, the difference between the algorithms for different LEO systems is also reduced, and good cooperative navigation and positioning performance can be obtained using different LEO constellations. These results show that the proposed algorithm is generally applicable to the future LEO constellation scheme of integrated communication and navigation (ICN).

### 5.2. Comparison with Other Typical Algorithms

To verify the performance of our algorithm, we horizontally compare the proposed algorithm with some other representative algorithms, including the noncooperative multi-aircraft positioning algorithm based on MEO constellation [48], non-cooperative LEO assistance the MEO multi-aircraft navigation and positioning algorithm [49], and the noncooperative multi-aircraft positioning algorithm-LEO constellation [16] (where Algorithm [16] represents a case of no altimeter assistance, and Algorithm + [16] represents the case of unbiased altimeter assistance). Our algorithm takes multi-aircraft cooperative navigation aided by an unbiased altimeter and multi-aircraft cooperative navigation without the aid of an altimeter for aircraft 1 as examples (corresponding to “this algorithm +” and “this algorithm”, respectively). The statistical results are shown in Table 3 and Table 4, where the algorithms in [48,49] do not include velocity indicators, so we do not provide comparative data for the velocity error indicators in Table 4.

From the comparison of position error statistics, even without the aid of altimeters, our proposed algorithm can achieve a performance similar to that of algorithm [48] and that of algorithm [49], and has a performance similar to that of algorithm [16] under noncooperative conditions. It also has a great performance advantage, and in addition to the down error, it can also achieve a performance similar to that of the altimeter-assisted Algorithm + [16]. However, with the aid of an altimeter, the performance of the multi-aircraft cooperative navigation and positioning algorithm based on the DWAPC-TSM system is obviously better than that of the previous algorithm, and the addition of an altimeter can significantly improve the ground position error.

From the perspective of velocity error, as a comparison of the same kind, the performance of the multi-aircraft cooperative navigation and positioning algorithm based on the DWAPC-TSM system is significantly better than that of the noncooperative multi-aircraft navigation and positioning algorithm without the aid of an altimeter, even with the altimeter-assisted noncooperative navigation and positioning algorithm. In addition to individual direction indicators, it still has certain advantages. When an altimeter is used, the performance of the multi-aircraft cooperative navigation and positioning algorithm based on the DWAPC-TSM system can be further improved.

## 6. Discussion, Conclusions and Future Works

In this paper, a multi-aircraft cooperative navigation and positioning algorithm based on the DWAPC-TSM system is proposed, and the ranging and time synchronization accuracy of the DWAPC-TSM system, as well as the corresponding algorithm structure and error analysis, are deduced and described in detail. To achieve high-precision time synchronization accuracy and ranging accuracy, based on this system, we propose a multi-aircraft collaborative navigation algorithm, adding the relative ranging and velocity measurement values obtained by the ranging and velocity sensor to the algorithm, and using this high-precision observation value to correct the absolute positioning observation value. Through the TWP-CTM system, the clock bias can be considered to be eliminated, under the following conditions:

(1) Whether it is an altimeter-free or altimeter-assisted scenario, it can effectively suppress the divergence of navigation and positioning results caused by pure INS applications, and can further improve the single-satellite navigation and positioning accuracy under coordination;
(2) Even if the formation performs cooperative navigation under different flight configurations and relative measurement accuracy, the algorithm can ensure good adaptability and robustness, and it can provide good location services which can be used as a reference scheme for feasibility exploration of a new cooperative navigation and positioning mode based on LEO communication satellites;
(3) Under different relative measurement accuracy, algorithm can still ensure good stability and robustness, indicating that the algorithm can be applied to flight business requirements equipped with sensors with different measurement accuracy, and is highly suitable for different application fields with cost requirements;
(4) Under different LEO constellations, the algorithm shows good universality, which means that the algorithm has strong scalability and adaptability for future ICN technical solutions, and can be used as a reference solution;
(5) Compared with other typical algorithms, our algorithm has specific advantages in various indicators, particularly for the absolute positioning situation of single satellite. It can be seen that adding relative measurement information can effectively improve the absolute positioning performance of formation members, which is a highly suitable a reference scheme for large-scale formation integration and collaborative control in the future.

However, since the LEO constellation has not been deployed globally and is not currently available for civilian use, and due to the current objective experimental conditions, implementation of the DWAPC-TSM system in engineering and the experimental verification of algorithm will be the direction of our future work. In addition, further improving the navigation and positioning accuracy will be our research focus in the future.

## Figures and Tables

**Table 1:** Main parameters of the Kuiper constellation and aircraft.

Constellation		Aircraft	
Orbital height	610 km	vAc1	approximately 1 Mach
Orbital inclination	42°	vAc2	approximately 1 Mach
Orbital surfaces	36	vAc3	approximately 1 Mach
Number of satellites per orbit	36	L	10 km
Total number of satellites	1296	h	5 km

**Table 2:** Main parameters of INS and relative ranging and velocity sensor.

Parameter	Value
$\mathrm{Gyro}\;\mathrm{noise}\;\mathrm{root}\;\mathrm{PSD}/({}^{\circ}/\sqrt{h}$)	0.002
Gyroscope first-order Markov noise RMS/(deg/$\sqrt{h}$)	0.002
Accelerometer noise root PSD/${\mu g}_{0}$	30
Accelerometer first-order Markov noise RMS/${\mu g}_{0}$	10
Relative distance measurement white noise RMS/m	0.2
Relative velocity measurement white noise RMS/($m\times s^{-1}$)	0.02
Data link ranging error/m	10

**Table 3:** Statistical comparison of position errors.

Algorithm	Mean (m)			STD (m)		
	N	E	D	N	E	D
Algorithm [48]	/	/	/	15.7	43.2	3.8
Algorithm [49]	−7.489	20.762	−140.377	3.659	1.218	28.266
Algorithm [16]	−32.6619	−41.9907	70.4270	21.4059	46.4180	79.3741
Algorithm + [16]	−6.6661	−9.5780	0.0008	7.8891	17.4749	0.0182
This algorithm	5.5416	4.7852	−32.6606	14.5480	6.4505	27.4803
This algorithm +	3.7988	8.3101	0.1775	2.7011	6.0367	0.2184

**Table 4:** Statistical comparison of velocity errors.

Algorithm	Mean (m/s)			STD (m/s)		
	N	E	D	N	E	D
Algorithm [16]	−0.1898	−0.6012	−0.5068	0.1139	0.6136	0.5080
Algorithm + [16]	−0.1385	−0.5263	−0.0004	0.1543	0.2787	0.0055
This algorithm	0.0191	0.0497	0.2152	0.2592	0.1560	0.1161
This algorithm +	0.0438	−0.2325	−0.0138	0.0850	0.1845	0.0183

## Data Availability

Not applicable.
